# Vaccinating girls, abandoning women: the financing asymmetry between HPV immunization and the cervical cancer treatment cascade in low- and middle-income countries

**DOI:** 10.3389/fonc.2026.1941545

**Published:** 2026-09-15

**Authors:** Shyamkumar Sriram

**Affiliations:** Department of Rehabilitation and Health Services, College of Public Affairs and Health Sciences, University of North Texas, Denton, TX, United States

**Keywords:** benefit package design, cervical cancer elimination, health financing, human papillomavirus, low- and middle-income countries, out-of-pocket expenditure, provider payment, universal health coverage

## Abstract

Cervical cancer is the first cancer for which the World Health Organization has adopted a global elimination strategy with explicit incidence and coverage targets, and that strategy rests on three pillars: vaccinate 90% of girls, screen 70% of women, and treat 90% of those found to have disease. Only the first is backed by a global pooled-procurement and co-financing mechanism. Screening, triage, treatment of precancer, and the management of invasive disease are financed nationally where they are financed at all, through general budgets, benefit packages, and time-limited donor projects, and where those fall short, they are paid for at the point of use. This Perspective argues that this gap is structural rather than incidental and that it produces a particular kind of unfairness. Vaccination protects a cohort that will not meet the disease for decades. The women dying of cervical cancer now were born too early to be vaccinated, and every remaining step of care carries a price. Recent changes moving vaccine financing into national budgets sharpen this problem rather than resolving it. Four remedies are proposed, each concerning what governments agree to buy and how they pay for it rather than how much external money arrives.

## 1 Introduction

In 2020, the World Health Assembly committed to eliminating cervical cancer as a public health problem. The commitment was unusually precise: elimination was defined as a national incidence below four cases per 100,000 women-years, and three interim targets were set for 2030: 90% of girls fully vaccinated against human papillomavirus (HPV) by 15 years of age, 70% of women screened with a high-performance test at 35 years and again at 45 years, and 90% of women found to have cervical disease treated (1). The modeling suggests that it can be met, with sustained achievement of all three targets averting tens of millions of cases and deaths across 78 low-income and lower-middle-income countries (2, 3).

Three terms recur below and are not interchangeable. Screening means any test offered to asymptomatic women, including cytology and visual inspection with acetic acid. A high-performance test means the HPV DNA or mRNA assay the 70% target specifies. Screen-and-treat means treating an eligible woman at the same visit rather than recalling her.

Six years in, the three pillars are not moving together. Vaccination has moved fast. Coverage across the lower-income countries supported by Gavi, the Vaccine Alliance, went from 4% in 2019 to 25% in 2024, helped by the single-dose schedule and second-phase introductions in large countries (4, 5). By late 2025, Gavi was reporting more than a million future deaths averted and some 86 million girls protected (6). India’s campaign in February 2026 offered free vaccination to approximately 11.5 million 14-year-old girls before the vaccine moved into the Universal Immunization Programme, the largest single HPV implementation effort attempted anywhere (7). Pakistan’s first national campaign, launched in September 2025, protected more than 9.6 million girls aged 9 to 14 in its opening phase, reaching 66% coverage (8).

The other two pillars have barely moved. Two in three women aged 30 to 49 worldwide have never been screened by any method, and coverage is thinnest where the disease is heaviest: country-level medians run at about 44% across low- and middle-income countries and approximately 17% in sub-Saharan Africa (9, 10). Radiotherapy, standard care for locally advanced disease, is scarce in exactly those countries, which hold a small share of the world’s teletherapy machines and a documented shortfall in brachytherapy (11, 12). The ordering is worth stating plainly: screening and cancer treatment services long predate the HPV vaccine, yet the youngest pillar has advanced fastest against its own target (**Figure 1B**).

**Figure 1:**
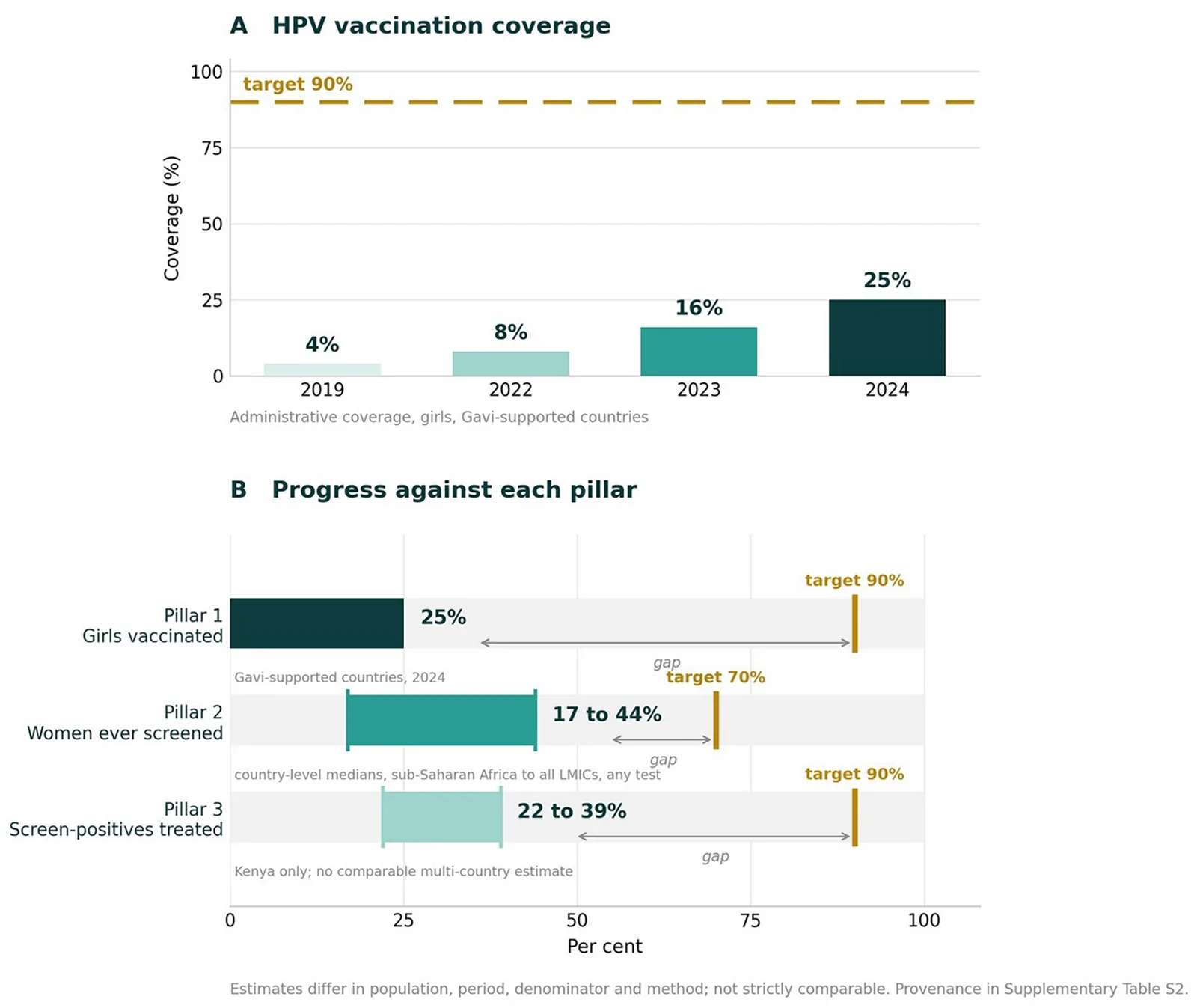
Progress against the three pillars of cervical cancer elimination. **(A)** HPV vaccine coverage among girls in Gavi-supported lower-income countries, 2019 to 2024, against the 2030 target of 90% (4). **(B)** Reported performance on each pillar against its own target. The three estimates come from different populations, periods, denominators, and data systems and are not strictly comparable: pillar 1 is administrative coverage in Gavi-supported countries in 2024; pillar 2 is the country-level median of self-reported lifetime screening by any method among women aged 30 to 49, shown as the range from sub-Saharan Africa to all low- and middle-income countries in surveys fielded 2005 to 2018 (10), which is a weaker test than the high-performance test the 70% target specifies; pillar 3 is the reported proportion of screen-positive women treated in Kenya (23, 24), shown as a single-country illustration because no comparable multi-country estimate exists. Full provenance for every plotted estimate, including numerators, denominators, countries, periods, estimation method, target definition, and data-quality limitations, is available from the author on request.

The usual explanation is that vaccination is simply easier to deliver than screening, and screening is easier than cancer treatment. That is true, but treating it as the whole story turns a financing question into a logistics one. Vaccination is the only pillar with a dedicated global pooled-procurement and co-financing mechanism behind it. The comparison drawn throughout is with instruments of that kind, and not with national financing, which does cover parts of the cascade in some countries. What follows is an argument that this asymmetry has a predictable and unfair result, that the current shift in vaccine financing worsens it, and that the remedy lies mostly in how governments purchase care.

## 2 One strategy, three pillars, one global financing instrument

Financing instrument, throughout this paper, means a dedicated internationally coordinated mechanism that pools procurement, negotiates a subsidized price, funds introduction, and commits governments to a multi-year co-financing schedule against a monitored coverage target. National budget lines, benefit packages, and time-limited donor projects are financing routes but not instruments in that sense.

Gavi’s HPV program deserves the credit it gets: it brought the per-dose price down far enough for national introduction to be realistic, paid introduction costs, and set up a co-financing path under which governments take on a growing share (4, 13). Forty-two countries had launched national programs with Gavi support by the end of 2024 (4). No instrument of that design exists for an HPV DNA test, thermal ablation or loop excision, cervical cancer surgery, or radiotherapy and brachytherapy. None of this means the vaccination pillar is finished. Coverage in Gavi-supported countries stood at 25% in 2024 against a 90% target (**Figure 1A**), and the co-financing transition described in Section 5 will test national budgets for a decade. The argument here is for extending instruments to the second and third pillars, not for withdrawing support from the first.

The claim needs care, because the cascade is not unfinanced everywhere. Ministries fund screening from general budgets, insurance covers cancer treatment in some countries, and donor projects have shaped prices and delivered services at scale: Unitaid and partners negotiated price reductions of about 40% for HPV tests, to under 9 US dollars, and about 45% for thermal ablation devices, introduced those devices in a fifth of all low- and middle-income countries, and committed a further 50 million US dollars in October 2025 (14, 15), and the Go Further partnership has delivered more than 10 million screens and over 400,000 precancer treatments for women living with HIV in 12 African countries (16, 17). These arrangements are project-scale and time-limited rather than replenishment-backed, eligibility often runs through HIV status rather than age, none carries a co-financing schedule, and they stop at precancer, since PEPFAR does not fund treatment of invasive disease (16). **Figure 1** shows how far each pillar stands from its own target, with caveats and provenance given in the caption.

Prices diverge first. Vaccine prices for eligible countries came down through pooled procurement and deliberate market shaping (13). No comparable global arrangement covers a colposcope, an ablation device, or a brachytherapy afterloader, all of which are bought through fragmented national procurement. A country can therefore buy HPV vaccine at a deeply subsidized pooled-procurement price while paying an unsubsidized market price for the HPV DNA test used to screen the older women the vaccine cannot help, because they were already past vaccination age when the programme began.

The decisions are then taken in different rooms. Vaccine financing sits inside the national immunization program, cushioned by an external co-financing commitment. Screening and treatment sit inside the general health budget and the benefit package, where cervical cancer takes its chances against every other claim. Nothing reconciles the two.

Whatever is left unfunded still gets paid for by somebody. A service that is neither publicly financed nor covered by a benefit package is paid for at the point of use, or it does not happen.

## 3 The arithmetic of the protected cohort

The central fact for financing design is that HPV vaccination protects a birth cohort, not a population, on a delay measured in decades (**Figure 2**).

**Figure 2:**
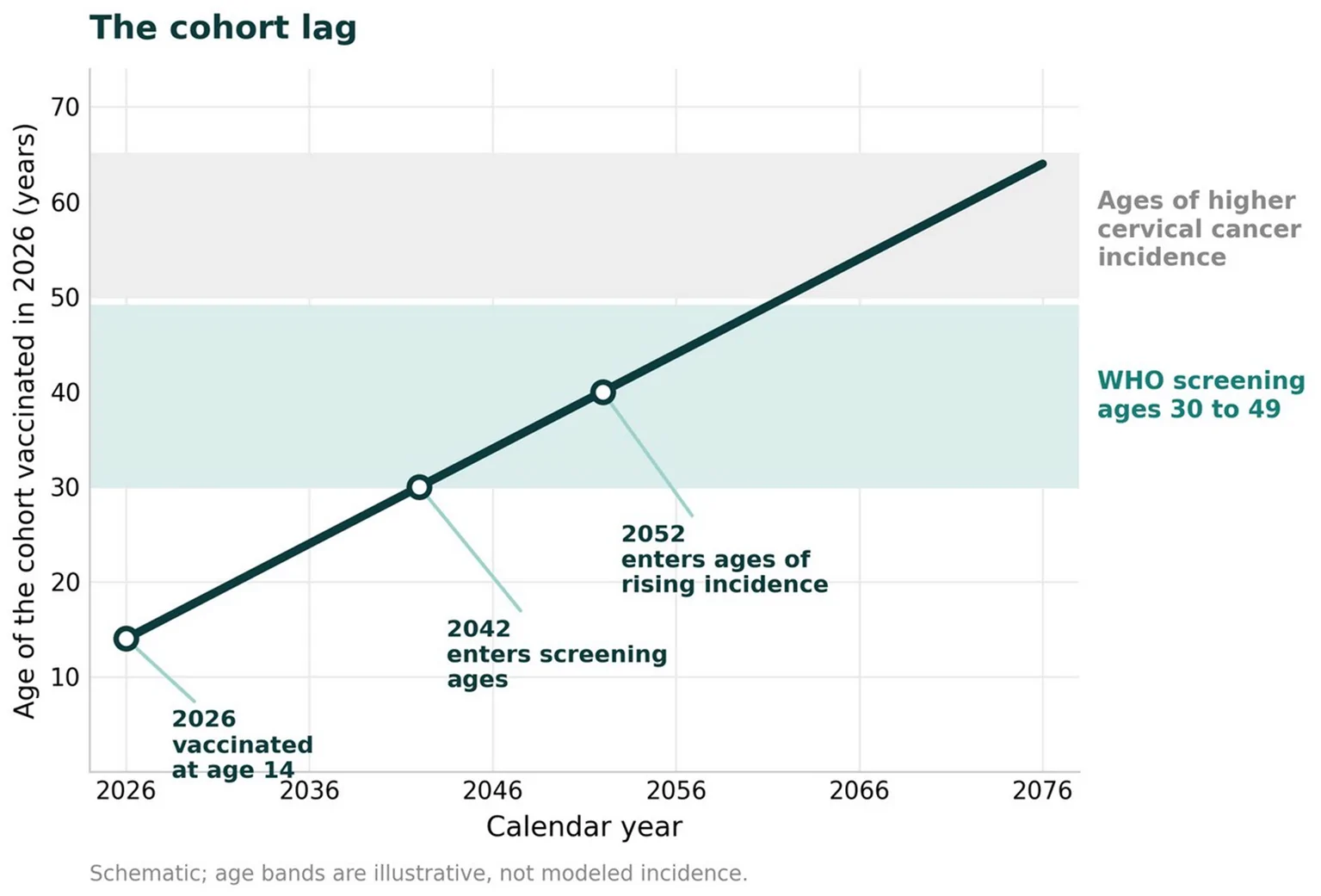
The cohort lag. The line traces the age of the cohort vaccinated at 14 years in 2026. Shaded bands mark the WHO screening ages and the ages of higher cervical cancer incidence, which the cohort reaches only in the 2040s and 2050s. Age bands are illustrative rather than modeled.

A girl vaccinated at 14 in 2026 reaches the ages of peak incidence in the 2050s and 2060s. Every woman who will be diagnosed with cervical cancer between now and mid-century was born before her country had a program to vaccinate her. For that entire generation, essentially every woman now past adolescence in most low- and middle-income countries, the only interventions available are the two that are least funded.

The modeling behind the strategy is explicit about the timing. In the WHO Cervical Cancer Elimination Modelling Consortium analyses of 78 low-income and lower-middle-income countries, girls-only vaccination, modeled as vaccination at 9 years with catch-up to 14, 90% coverage, and no change to screening or treatment, would reduce cervical cancer mortality by 0.1% by 2030 against the status quo, and by 61.7% only by 2070. Adding twice-lifetime HPV testing at 35 and 45 with scale-up of cancer treatment would reduce mortality by 34.2% by 2030, averting approximately 300,000 deaths (2, 3). These are modeled scenario outputs under assumed coverage trajectories rather than observed effects. Read that way, the near-term dividend belongs to the two pillars without a dedicated global instrument, and the long-term one belongs to the funded pillar.

Global health has thus concentrated its money on the intervention with the longest payback and left to household budgets the interventions that decide how many women die in the next 20 years. That is defensible on discounted cost-effectiveness grounds, but not on equity grounds, and not what governments signed up to in 2020.

## 4 The priced cascade and its equity inversion

Follow a woman in a lower-middle-income country through a system where the vaccine is free and nothing after it is. She pays to reach the clinic, for the screening test, for triage, for ablation or excision of a precancerous lesion, and for histopathology, staging imaging, surgery, chemotherapy, radiotherapy and brachytherapy, and, eventually, palliative care. She loses income at every step, as does whoever travels with her.

The evidence on household impact is consistent. Cancer care in India generates high out-of-pocket spending, diagnostics dominating outpatient costs and medicines dominating inpatient costs, and a large share of affected households borrow or sell assets to meet it (18, 19). A longitudinal cohort of patients with breast cancer in Mumbai found catastrophic expenditure and distress financing at strikingly high rates even among women who had some reimbursement (20), and cross-national work shows catastrophic spending still rising across many low- and middle-income countries despite coverage expansion (21). Hardship of this kind is not a side effect of the cascade. It is how the cascade sheds patients, because a woman who cannot pay stops coming, and a curable cancer becomes a fatal one.

The unfairness is by now visible: what is free goes to adolescent girls not yet at risk, what is priced goes to adult women at risk today, who are disproportionately poor, rural, and less educated.

The gradients also differ. Vaccination reaches girls through schools and routine immunization; screening requires an adult woman to travel, forgo work, and usually to pay. Free vaccination therefore flattens the adolescent gradient while priced screening steepens the adult one. Inequalities in screening coverage within low- and middle-income countries are well documented and have proved stubborn (10, 22). A country’s headline vaccination figure can improve while its screening gradient worsens, and nothing in current monitoring would show it.

### 4.1 The clinical consequence: attrition, stage, and treatment time

Household payment reaches the tumor by three routes: whether a screen-positive woman is treated at all, how advanced her disease is when it is found, and whether her radiotherapy finishes on time.

Attrition between a positive screen and treatment is where the cascade breaks most often. Kenya has had a single-visit screen-and-treat policy since 2013, yet screening uptake has been put at approximately 16%, and only about one-fifth to two-fifths of women found to have precancer are reported to have been treated (23, 24). Ethiopian work documents the same pattern in follow-up after a positive screen, and the reasons women give are informational, geographical, and financial rather than clinical (25). A precancer found and not treated is a diagnosis with no therapeutic act attached: the woman goes on to invasive disease having been identified by the system that then failed to treat her. Any step that adds a fee and a second visit sheds the poorest women first, which is why WHO guidance prefers same-visit ablative treatment wherever a woman is eligible for it (26).

Stage at presentation is the second route. Where screening is unaffordable, cancer announces itself through symptoms rather than detection, and the problem changes character, from an ablative procedure a nurse can perform at the primary level to locally advanced disease needing chemoradiotherapy and brachytherapy at a tertiary center. The cost difference is not marginal, and it lands on the same household that could not afford the cheaper option.

The third route is one radiation oncologists will recognize and health financing rarely mentions. Outcome in locally advanced disease depends on overall treatment time as well as on dose. Prolongation reduces local control by roughly 0.3% to 1.6% for each additional day, through accelerated repopulation, and cohort data put the loss at approximately 0.7% in pelvic control and 0.6% in beyond 55 days (27, 28). Cohort evidence accordingly supports completing external beam radiotherapy and brachytherapy within about 8 weeks (29). Now, picture a woman paying out of pocket for transport, lodging, imaging, or brachytherapy consumables who interrupts treatment for a fortnight while the money is raised. That is not an administrative delay but a measurable reduction in her chance of cure, produced by a radiobiological rather than a bureaucratic mechanism.

A corollary follows for community engagement, the usual instrument for closing the information gap. The Ethiopian barriers were informational, geographical, and financial together rather than informational alone (25). Community mobilization, peer navigation, and outreach act on the first of those, and none acts on the third. Where the cascade is publicly financed, moving women into it is the intended result. Where it is not, successful engagement produces informed women who present, are screened, are told they have a precancerous lesion, and then cannot pay for the ablation. Demand generation and cascade financing are complements rather than alternatives.

This is why the asymmetry is an oncology problem and not only a health systems one. The dose is prescribed by the radiation oncologist; the gap between fractions is too often prescribed by the household budget.

## 5 The transition in vaccine financing

Over the current strategic period, HPV vaccination in Gavi-supported countries is being paid for through national vaccine budgets for the first time, with most procurement decisions handed to governments so that they can prioritize according to their own strategy and circumstances (30). Gavi has said openly that countries will face choices with real consequences for lives saved (30).

There is a good case for the reform: it gives governments genuine ownership and a multi-year view of their resources. On the specific problem described here, though, it makes things harder, for three reasons.

It turns HPV from a protected line into a contested one, arguing its case inside the vaccine budget against pneumococcal, rotavirus, measles-rubella, typhoid conjugate, and malaria vaccines - and doing so with the longest payback period of any of them. In a tight budget judged on deaths averted soon, a vaccine whose benefits land in the 2050s is at a structural disadvantage.

It also does nothing for the other two pillars. Handing over procurement decisions reallocates authority; it does not create fiscal space for HPV DNA testing or for a linear accelerator.

The transition also arrives while development assistance for health is contracting, so the space these decisions are devolved into is shrinking as the decisions become national ones.

None of which is an argument for reversing it, only that owning the vaccination pillar makes sense if a country also finances the other two, and that the transition ought to trigger purchasing reform rather than substitute for it.

## 6 Discussion

Four remedies follow, all things a health ministry or national purchaser can do, none requiring a new global financing instrument.

Put screen-and-treat in the statutory benefit package. For most governments, this costs no new money; it changes what publicly financed insurance agrees to buy. A scheme that pays for cervical cancer surgery, chemotherapy, and radiotherapy but not for HPV testing, triage, and ablation of precancer is buying the expensive consequence of the cheap omission. Covering primary HPV testing and same-visit treatment turns a late-stage, high-cost, low-survival pathway into an early-stage, low-cost, high-survival one.

Pay for the episode rather than the test. Fee-for-service breaks the cascade into separately reimbursed pieces and imposes no cost on a provider when a screen-positive woman never returns, so screening becomes revenue whether or not treatment happens. A bundled rate covering screening, triage, and precancer treatment as one episode ties the provider’s income to whether women finish rather than to how many tests are run. A completion condition needs a guard against providers avoiding the women hardest to needs a guard - risk adjustment or an exclusion floor - against providers avoiding the women hardest to follow up.

Earmark health tax revenue for the cascade, not only for doses. Tobacco, alcohol, and sugar taxes are increasingly used across low- and middle-income countries to raise revenue with a health benefit attached. Earmarked revenue can be offset elsewhere, but where the practice is politically possible, a defined share sent to screening and precancer treatment funds the part of the strategy that nothing else funds.

Measure completion, not coverage. What gets measured gets funded. Vaccination coverage is reported annually and belongs to an accountable partnership. Screening coverage is reported irregularly, treatment completion rarely, and household spending along the cascade almost never. A framework reporting by country, split by wealth quintile and residence, what proportion of screen-positive women finish treatment, and what proportion of households are ruined paying for it would put the asymmetry in front of the people who write budgets.

The argument has limits worth stating. It is a claim about global financing architecture rather than an estimate of its cost in lives; the obvious counterfactual, how many deaths follow from absent cascade financing rather than absent capacity, is not quantified here. A reader may reasonably reply that vaccination attracts the instrument because it is the most cost-effective of the three. The evidence is not that tidy: in the WHO Eastern Mediterranean Region investment case, cervical cancer treatment returns more per dollar than vaccination in the lowest-income country group, while screening returns below a dollar in every group, a figure the report flags as a probable underestimate (31). The pillar with the strongest modeled return in the poorest settings is the one with no instrument at all. In any case, that objection concerns allocative efficiency, while the claim here is distributional. The remedies also assume a purchaser capable of acting, which not every low-income health system has.

The strategy commits governments to three numbers. The global financing architecture funds one. The result is an inversion: the free intervention protects women not yet at risk while the priced ones fall on women dying now, with household payment as funder of last resort for two-thirds of the elimination effort. Elimination was never a vaccination target with two aspirations attached. It is a financing problem with three numbers in it, and only one of them has a global instrument behind it.

## Data Availability

The original contributions presented in the study are included in the article/supplementary material. Further inquiries can be directed to the corresponding author.
